# Brain hyperintensities in magnetic resonance imaging of patients with mild acute focal neurology

**DOI:** 10.1007/s10072-020-04256-1

**Published:** 2020-01-23

**Authors:** Ourania Varsou, Katie Turnbull, Michael S Stringer, Catarina Dinis Fernandes, Alison D Murray, Christian Schwarzbauer, Mary Joan Macleod

**Affiliations:** 1 Anatomy Facility, School of Life Sciences, University of Glasgow, Glasgow, UK; 2 Brain Research Imaging Centre, Centre for Clinical Brain Sciences, University of Edinburgh, Edinburgh, UK; 3 UK Dementia Research Institute at the University of Edinburgh, Edinburgh Medical School, Edinburgh, UK; 4 The Netherlands Cancer Institute, Amsterdam, The Netherlands; 5 Aberdeen Biomedical Imaging Centre, University of Aberdeen, Aberdeen, UK; 6 Faculty of Applied Sciences & Mechatronics, Munich University of Applied Sciences, Munich, Germany; 7 The Institute of Medical Sciences, University of Aberdeen, Aberdeen, UK

**Keywords:** White matter hyperintensities, Leukoaraiosis, Stroke, TIA, Migraine

## Abstract

**Purpose:**

Hyperintensities are common in neuroimaging scans of patients with mild acute focal neurology. However, their pathogenic role and clinical significance is not well understood. We assessed whether there was an association between hyperintensity score with diagnostic category and clinical assessments/measures.

**Methods:**

One hundred patients (51 ± 12 years; 45:55 women:men), with symptomatology suggestive of short duration ischemia referred for magnetic resonance imaging, were prospectively recruited in NHS Grampian between 2012 and 2014. Hyperintensities were quantified, on T_2_ and FLAIR, using the Scheltens score.

**Results:**

The most frequent diagnosis was minor stroke (33%), migraine (25%) and transient ischemic attack (17%). The mean total Scheltens score was 28.49 ± 11.93 with all participants having various loads of hyperintensities. Statistically significant correlations between hyperintensity scores and clinical assessments/measures (age, systolic blood pressure, pulse pressure, MoCA) at the global level were also reflected regionally. These provide further supporting data in terms of the robustness of the Scheltens scale.

**Conclusion:**

Hyperintensities could serve as a diagnostic and prognostic imaging biomarker for patients, presenting with mild acute focal neurology, warranting application of automated quantification methods. However, larger cohorts are required to provide a definitive answer especially as this is a heterogenous group of patients.

**Electronic supplementary material:**

The online version of this article (10.1007/s10072-020-04256-1) contains supplementary material, which is available to authorized users.

## Introduction

White matter hyperintensities are frequently observed in neuroimaging structural scans of patients with mild acute focal neurology who are later diagnosed with minor stroke, transient ischemic attack (TIA), migraine or a functional neurological disorder. However, their underlying pathogenic role and clinical significance [1, 2], especially in this patient group (or type), has not been fully explained. A better insight into this has the potential not only to aid the diagnostic process and immediate management plans, considering the challenges for diagnosing patients with mild acute focal neurology, but also to inform risk predictor models of recurring focal neurology along with quantifying future functional and cognitive outcomes.

The Scheltens scale is a visual rating scale that describes the anatomical distribution of hyperintensities and quantifies their amount (total number and size of each hyperintensity) providing information at the global and regional levels [3, 4]. We assessed whether there was a difference in the total Scheltens score and different diagnostic categories along with ascertaining the observed power to inform future study designs. We also assessed whether there was an association between total and individual hyperintensity scores—and various clinical assessments/measures as described in the Supplementary File. The purpose was to explore whether statistically significant results relating to global changes were reflected at a regional level with any patterns of clinical relevance.

## Methods

One hundred patients, with a mean age of 51 ± 12 years and a 45:55 women to men ratio, with mild acute focal neurology consistent with a diagnosis of short duration ischemia [5, 6] referred for magnetic resonance imaging (MRI) were prospectively enrolled in NHS Grampian between 2012 and 2014. Imaging data were acquired on a 3.0 Tesla Philips Achieva scanner (Philips Healthcare, Best, The Netherlands; http://www.philips.com/global/index.page) with a Siemens 32-channel coil (Siemens Medical Systems, Iselin, NJ; http://www.healthcare.siemens.co.uk). Hyperintensities were assessed on axial T_2_ and FLAIR scans, by a blinded assessor, using the Scheltens scale [3, 4].

A one-way ANOVA assessed for any significant differences in the Scheltens score and different diagnostic categories. The Pearson’s explored the correlations between the Scheltens scores and various clinical assessments/measures. The Benjamin-Hochberg procedure was applied, with a false discovery rate (FDR) of 0.05, to control for type I errors (false positives) potentially arising from multiple comparisons. Analysis was performed in SPSS version 25 (IBM Corporation, Armonk, NY; http://www-01.ibm.com/software/analytics/spss/). Full methodological details are available in the Supplementary File.

## Results

The most frequent diagnosis was minor stroke (33%), followed by migraine (25%), TIA (17%) and functional neurological disorders (7%). The remaining 18% were diagnosed as ‘other’ with a full list provided in the Supplementary File. The ANOVA yielded no statistically significant differences in the total Scheltens scores and different diagnostic categories (*F*[4,95] = 1.177, exact *p* = 0.326, Adjusted R Squared = 0.007). However, the observed power for this part of the analysis was 36%. The mean total Scheltens score was 28.49 ± 11.93 with all participants having various loads of hyperintensities, both globally and regionally, but with no one having zero hyperintensities at any level (Table 1).

**Table 1:** Descriptive statistics of clinical assessments and measures*

Assessment/measure	Mean ± SD	95% lower–upper CI	Min-Max range
Systolic blood pressure	128.33 ± 17.71	124.75–131.92	98–200
Diastolic blood pressure	80.21 ± 10.65	78.05–82.37	50–119
Pulse pressure	48.13 ± 13.03	45.48–50.77	17–94
Modified Rankin Scale (0–6)	0.30 ± 0.61	0.18–0.42	0–4
NIHSS (/42)	0.32 ± 0.73	0.18–0.47	0–3
MoCA (/30)	28.61 ± 1.54	28.27–28.95	23–30
Risk factors score (0–5)**	0.72 ± 1.09	0.50–0.94	0–4
Total Scheltens score (/93)	28.49 ± 11.93	26.12–30.86	6–73
White matter hyperintensities (/30)	9.44 ± 5.94	8.26–10.62	1–30
Periventricular hyperintensities (/9)	4.41 ± 1.66	4.08–4.74	1–9
Gray matter hyperintensities (/30)	8.27 ± 3.86	7.50–9.04	1–21
Infratentorial hyperintensities (/24)	6.36 ± 3.22	5.72–7.00	1–15

*Recorded, and not log transformed, values reported in table

**Calculated from the following past medical history questions: hypertension; hyperlipidemia; diabetes mellitus; ischemic heart disease; and previous TIA or stroke, with a point was awarded for ‘yes’ answers

*CI*, confidence interval; *SD*, standard deviation

With the Benjamini-Hochberg post hoc correction, the following statistically significant findings emerged. A positive correlation between: (i) age with total Scheltens score (*r* = 0.550; *p* < 0.001), white matter (*r* = 0.585; *p* < 0.001), periventricular (*r* = 0.467; *p* < 0.001) and gray matter hyperintensities (*r* = 0.399; *p* < 0.001); (ii) systolic blood pressure with total Scheltens score (*r* = 0.340; *p* = 0.001), white (*r* = 0.309; *p* = 0.002) and gray matter hyperintensities (*r* = 0.355; *p* < 0.001); (iii) pulse pressure with total Scheltens score (*r* = 0.325; *p* = 0.001), white matter (*r* = 0.352; *p* < 0.001), periventricular (*r* = 0.277; *p* = 0.006), and gray matter hyperintensities (*r* = 0.245; *p* = 0.016); (iv) diastolic blood pressure with gray matter hyperintensities (*r* = 0.243; *p* = 0.017); and (v) cumulative risk factor score with periventricular hyperintensities (*r* = 0.271; *p* = 0.006). A negative correlation between the MoCA (MoCaTest, Québec, Canada, https://www.mocatest.org) and the total Scheltens score (*r* = − 0.280; *p* = 0.012) and white matter hyperintensities (*r* = − 0.300; *p* = 0.007) was also observed.

## Discussion

This study provides further data about the potential role of hyperintensities in patients with mild acute focal neurology. Although there were no statistically significant differences in the total Scheltens score and the different diagnostic categories, it is still of clinical importance that all participants had hyperintensities on their scans with a minimum total score of six and no individual Scheltens scale components having a zero score (Table 1). The observed power also suggests that larger cohorts are needed to provide a definitive answer as to whether different diagnoses are associated with different hyperintensity loads.

The significant positive correlation between the total Scheltens score and age is not unexpected, as hyperintensities become more prevalent with increasing age [1]. However, this global change was also noted regionally with a significant positive correlation in age and amount of hyperintensities within the white and gray matter and around the ventricles. The significant negative correlation between MoCA and the total Scheltens score and white matter scores supports published literature linking hyperintensities with declining cognitive function [1]. The links between systolic blood pressure and pulse pressure with the total Scheltens score and some of its individual components provide additional information that these parameters might be involved in their pathogenesis. However, this is not a straightforward cause-effect relationship and research suggests that genes, which regulate a variety of biological processes, also play a contributing part [2]. These results should be interpreted with caution, as this is a heterogenous group of patients with each diagnostic category having different pathophysiological mechanisms. The correlations were also between hyperintensity scores and recognised risk factors (age, hypertension) or outcomes (cognition) and the neurological presentation/diagnosis could have been incidental.

Although the Scheltens scale is a labour-intensive visual scale, the above findings showed that significant results in relation to the total score (global changes) were also reflected at the regional level in individual components of the scale. These provide further supporting data in terms of its robustness when compared with alternatives. Generally, manual assessment of hyperintensities is not perfect with ceiling effects and staff/training requirements being potential issues.

Hyperintensities could serve in the future as an additional imaging biomarker in mild acute focal neurology. However, further research is required, with larger cohorts, to determine their exact role in each diagnostic category and automated quantification will undoubtedly help with standardisation.

## Electronic supplementary material

ESM 1 (DOCX 117 kb).
