# Supplementary material for: Brain hyperintensities in magnetic resonance imaging of patients with mild acute focal neurology
Source: Neurol Sci. 2020 Jan 23;41(6):1633–5. doi: 10.1007/s10072-020-04256-1 (PMC7275935; doi:10.1007/s10072-020-04256-1)
Supplement: Supplementary file 1 — (DOCX 117 kb). [file 10072_2020_4256_MOESM1_ESM.docx]

**Hyperintensities in Mild Acute Focal Neurology**

**Supplementary File**

**Aims**

1. Assess whether there was a difference in the total Scheltens score for patients with different final diagnoses (i.e. minor stroke, TIA, migraine, functional neurological disorder, other) and separately explore whether sex played a role.* A secondary aim was to ascertain the observed power, for this part of the analysis, to allow for appropriate interpretation of the results and also inform future study designs.

*Note: The final diagnosis was made by the stroke consultant or senior registrar responsible for each patient.

1. Assess whether there was an association between the total Scheltens score and the following variables: sBP; dBP; pulse pressure; mRS; NIHSS; MoCA; cumulative score of five risk factors; age. A secondary aim was to assess whether there was an association between each individual component of the Scheltens scale (i.e. WMH, GMH, PVH, IFTH) and the above list of variables. The purpose of this was to explore whether statistically significant results in relation to the total Scheltens score (i.e. global changes) were also reflected at the regional level with the individual components of the Scheltens scale and identify any patterns of clinical relevance.

**Methods**

*Ethical Approval*

Approval for this study was granted by the North of Scotland Research Ethics Committee (reference number: 11/NS/0030). The study was also registered with the NHS Grampian Research and Development Department (reference number: 2011ST003). Written informed consent was obtained, from all study participants, prior to taking part in any research activity.

*Study Design*

This was a prospective neuroimaging study with patients referred from the NHS Grampian neurovascular clinic or the Acute Stroke Unit, between 2012 and 2014, with acute focal minor neurological symptoms consistent with a possible diagnosis of short duration ischemia (Easton *et al.*, 2009; Fischer *et al.*, 2010) for whom MRI would have been the investigation of choice. In terms of exclusion criteria, apart from standard MRI contraindications, the following also applied: i) < 18 years; ii) hemorrhagic stroke; iii) chronic mental health or neurodegenerative condition and brain tumours; and iv) moderate to severe carotid artery stenosis from doppler ultrasound. A detailed list of the inclusion and exclusion criteria is available by Varsou *et al.* (2014).

*Scanning Protocol*

Imaging data were acquired on the Aberdeen Biomedical Imaging Centre 3.0 Tesla Philips Achieva X-series MRI scanner (Philips Healthcare, Best, The Netherlands; <http://www.philips.com/global/index.page>) with a Siemens 32-channel receive-only phased-array head coil (Siemens Medical Systems, Iselin, NJ; <http://www.healthcare.siemens.co.uk>). The structural sequences had the following parameters:

1. axial T_2_-weighted short-τ inversion recovery spin echo structural sequence with a total acquisition time of 3 minutes and 6 seconds (repetition time of 3000 ms, echo time of 80 ms, inversion time τ of 100-150 ms, flip angle of 90˚, 230 × 184 × 129 mm^3^ field of view, 0.8 × 0.8 mm^2^ voxel size, and 26 slices);
2. axial FLAIR spin echo structural sequence with a total acquisition time of 5 minutes and 52 seconds (repetition time of 11000 ms, echo time of 125 ms, refocusing angle of 120˚, 230 × 230 × 144 mm^3^ field of view, 0.7 × 0.9 mm^2^ voxel size, and 29 slices).

*Scoring of Hyperintensities*

The signal hyperintensities were assessed on axial T_2_ and FLAIR MRI structural scans, provided by NHS Grampian PACS, using the Scheltens semiquantitative visual scoring scale by Murray *et al.* (2012). The WMH and PVH were assessed on FLAIR, whereas the GMH and IFTH were assessed on T_2_. This method has a good interobserver and intraobserver reliability for total scores when compared to alternative scales (Murray, 2012). The Scheltens scale also quantifies the number and size of lesions within each of the different anatomical areas providing information not only at the global, but also the regional level (Scheltens *et al.*, 1993; Scheltens *et al.*, 1998). A trained assessor (OV), who was blinded to the patients’ diagnosis at the time of the scoring, assessed the scans using the Scheltens scale. Additional information about the methodology is available in Murray *et al.* (2012) and Varsou *et al.* (2015).

*Cumulative Risk Factors Score*

A cumulative risk factors score was calculated from the following past medical history questions: i) hypertension; ii) hyperlipidemia; iii) diabetes mellitus; iv) ischemic heart disease; and v) previous TIA or stroke. A point was awarded for ‘yes’ answers to each of the above with the potential minimum to maximum range being 0 to 5.

*Normality Test & Log Transformations*

The Shapiro-Wilk test was used to assess normality for all numerical variables. Any variable, which was not normally distributed, was subsequently log transformed to base 10 (i.e. common log). The constant number ‘3’ was also added to any variable that had values of 0, as it is not possible to take the log of 0. Details of the normality tests and log transformations are included in the table below.

| **Variable** | **Shapiro-Wilk** | **Log Transformation** |
| --- | --- | --- |
| Scheltens score | *W* (100) = 0.959, p=0.004 | Lg10 (Scheltens score) |
| WMH | *W* (100) = 0.932, p<0.001 | Lg10 (WMH) |
| GMH | *W* (100) = 0.971, p=0.028 | Lg10 (GMH) |
| PVH | *W* (100) = 0.868, p<0.001 | Lg10 (PVH) |
| IFTH | *W* (100) = 0.972, p=0.029 | Lg10 (IFTH) |
| sBP | *W* (96) = 0.929, p<0.001 | Lg10 (sBP) |
| dBP | *W* (96) = 0.926, p<0.001 | Lg10 (dBP) |
| PP | *W* (96) = 0.945, p=0.001 | Lg10 (PP) |
| mRS | *W* (100) = 0.525, p<0.001 | Lg10 (mRS+3) |
| NIHSS | *W* (99) = 0.503, p<0.001 | Lg10 (NIHSS+3) |
| MoCA | *W* (80) = 0.812, p<0.001 | Lg10 (MoCA) |
| risk factors score | *W* (100) = 0.695, p<0.001 | Lg10 (RF+3) |
| age | *W* (100) = 0.980, p=0.138 | not log transformed |

*Statistical Analysis*

A one-way ANOVA was used to assess for any significant differences between the total Scheltens scores and the different diagnoses. The observed power of this statistical test was also calculated. For the ANOVA, p values of < 0.05 were accepted as statistically significant. The Pearson’s correlation coefficient was used to assess whether there was an association between the total/individual Scheltens scores and the various physical measurements/clinical assessments. The above analysis was performed in SPSS version 25 (IBM Corporation, Armonk, NY; <http://www-01.ibm.com/software/analytics/spss/>) by two independent researchers (OV & KT), who crosschecked all results to ensure no errors. To control for type I error (i.e. false positives) resulting from the multiple correlations, the Benjamini-Hochberg FDR procedure (Benjamini and Hochberg, 1995) was applied by an independent researcher (MS) as described in later parts of this supplementary file.

**Descriptive Statistics**

| **Descriptives** | | | | |
| --- | --- | --- | --- | --- |
|  | | | Statistic | Std. Error |
| **Age (years)** | Mean | | 50.95 | 1.202 |
|  | 95% Confidence Interval for Mean | Lower Bound | 48.56 |  |
|  |  | Upper Bound | 53.34 |  |
|  | 5% Trimmed Mean | | 51.14 |  |
|  | Median | | 51.50 |  |
|  | Variance | | 144.533 |  |
|  | Std. Deviation | | 12.022 |  |
|  | Minimum | | 21 |  |
|  | Maximum | | 82 |  |
|  | Range | | 61 |  |
|  | Interquartile Range | | 16 |  |
|  | Skewness | | -.287 | .241 |
|  | Kurtosis | | -.313 | .478 |

| **Sex** | | | | | |
| --- | --- | --- | --- | --- | --- |
|  | | Frequency | Percent | Valid Percent | Cumulative Percent |
| Valid | Male | 55 | 55.0 | 55.0 | 55.0 |
|  | Female | 45 | 45.0 | 45.0 | 100.0 |
|  | Total | 100 | 100.0 | 100.0 |  |

| **Final diagnosis** | | | | | |
| --- | --- | --- | --- | --- | --- |
|  | | Frequency | Percent | Valid Percent | Cumulative Percent |
| Valid | TIA | 17 | 17.0 | 17.0 | 17.0 |
|  | Minor stroke | 33 | 33.0 | 33.0 | 50.0 |
|  | Migraine | 25 | 25.0 | 25.0 | 75.0 |
|  | Non-organic | 7 | 7.0 | 7.0 | 82.0 |
|  | Other | 18 | 18.0 | 18.0 | 100.0 |
|  | Total | 100 | 100.0 | 100.0 |  |

| **Clarification of final diagnosis if other** | | | | | |
| --- | --- | --- | --- | --- | --- |
|  | | Frequency | Percent | Valid Percent | Cumulative Percent |
| Valid |  | 82 | 82.0 | 82.0 | 82.0 |
|  | Acute vestibular neuronitis | 1 | 1.0 | 1.0 | 83.0 |
|  | Anxiety | 2 | 2.0 | 2.0 | 85.0 |
|  | Ballismus | 1 | 1.0 | 1.0 | 86.0 |
|  | Global transient amnesia | 1 | 1.0 | 1.0 | 87.0 |
|  | Left ulnar neuropathy | 1 | 1.0 | 1.0 | 88.0 |
|  | Meniere's disease | 1 | 1.0 | 1.0 | 89.0 |
|  | Minor contusion or focal seizure | 1 | 1.0 | 1.0 | 90.0 |
|  | Neuropraxia | 1 | 1.0 | 1.0 | 91.0 |
|  | Partial seizure | 1 | 1.0 | 1.0 | 92.0 |
|  | Sporadic CJD | 1 | 1.0 | 1.0 | 93.0 |
|  | Stress | 2 | 2.0 | 2.0 | 95.0 |
|  | Subacute cerebellar infarct and migraine | 1 | 1.0 | 1.0 | 96.0 |
|  | Unclear | 4 | 4.0 | 4.0 | 100.0 |
|  | Total | 100 | 100.0 | 100.0 |  |

| **Descriptives** | | | | |
| --- | --- | --- | --- | --- |
|  | | | Statistic | Std. Error |
| **Sum of risk factors** | Mean | | .72 | .109 |
|  | 95% Confidence Interval for Mean | Lower Bound | .50 |  |
|  |  | Upper Bound | .94 |  |
|  | 5% Trimmed Mean | | .61 |  |
|  | Median | | .00 |  |
|  | Variance | | 1.194 |  |
|  | Std. Deviation | | 1.092 |  |
|  | Minimum | | 0 |  |
|  | Maximum | | 4 |  |
|  | Range | | 4 |  |
|  | Interquartile Range | | 1 |  |
|  | Skewness | | 1.387 | .241 |
|  | Kurtosis | | .812 | .478 |

| **Descriptives** | | | | |
| --- | --- | --- | --- | --- |
|  | | | Statistic | Std. Error |
| **Systolic blood pressure (mmHg)** | Mean | | 128.33 | 1.807 |
|  | 95% Confidence Interval for Mean | Lower Bound | 124.75 |  |
|  |  | Upper Bound | 131.92 |  |
|  | 5% Trimmed Mean | | 127.34 |  |
|  | Median | | 125.00 |  |
|  | Variance | | 313.467 |  |
|  | Std. Deviation | | 17.705 |  |
|  | Minimum | | 98 |  |
|  | Maximum | | 200 |  |
|  | Range | | 102 |  |
|  | Interquartile Range | | 20 |  |
|  | Skewness | | 1.103 | .246 |
|  | Kurtosis | | 2.210 | .488 |

| **Descriptives** | | | | |
| --- | --- | --- | --- | --- |
|  | | | Statistic | Std. Error |
| **Diastolic blood pressure (mmHg)** | Mean | | 80.21 | 1.087 |
|  | 95% Confidence Interval for Mean | Lower Bound | 78.05 |  |
|  |  | Upper Bound | 82.37 |  |
|  | 5% Trimmed Mean | | 79.73 |  |
|  | Median | | 80.00 |  |
|  | Variance | | 113.367 |  |
|  | Std. Deviation | | 10.647 |  |
|  | Minimum | | 50 |  |
|  | Maximum | | 119 |  |
|  | Range | | 69 |  |
|  | Interquartile Range | | 15 |  |
|  | Skewness | | .827 | .246 |
|  | Kurtosis | | 2.541 | .488 |

| **Descriptives** | | | | |
| --- | --- | --- | --- | --- |
|  | | | Statistic | Std. Error |
| **Pulse pressure (sBP-dBP; mmHg)** | Mean | | 48.13 | 1.330 |
|  | 95% Confidence Interval for Mean | Lower Bound | 45.48 |  |
|  |  | Upper Bound | 50.77 |  |
|  | 5% Trimmed Mean | | 47.53 |  |
|  | Median | | 45.00 |  |
|  | Variance | | 169.816 |  |
|  | Std. Deviation | | 13.031 |  |
|  | Minimum | | 17 |  |
|  | Maximum | | 94 |  |
|  | Range | | 77 |  |
|  | Interquartile Range | | 18 |  |
|  | Skewness | | .772 | .246 |
|  | Kurtosis | | 1.190 | .488 |

| **Descriptives** | | | | |
| --- | --- | --- | --- | --- |
|  | | | Statistic | Std. Error |
| **Modified Rankin Scale (0-6)** | Mean | | .30 | .061 |
|  | 95% Confidence Interval for Mean | Lower Bound | .18 |  |
|  |  | Upper Bound | .42 |  |
|  | 5% Trimmed Mean | | .22 |  |
|  | Median | | .00 |  |
|  | Variance | | .374 |  |
|  | Std. Deviation | | .611 |  |
|  | Minimum | | 0 |  |
|  | Maximum | | 4 |  |
|  | Range | | 4 |  |
|  | Interquartile Range | | 1 |  |
|  | Skewness | | 2.977 | .241 |
|  | Kurtosis | | 13.043 | .478 |

| **Descriptives** | | | | |
| --- | --- | --- | --- | --- |
|  | | | Statistic | Std. Error |
| **NIHSS (/42)** | Mean | | .32 | .073 |
|  | 95% Confidence Interval for Mean | Lower Bound | .18 |  |
|  |  | Upper Bound | .47 |  |
|  | 5% Trimmed Mean | | .20 |  |
|  | Median | | .00 |  |
|  | Variance | | .527 |  |
|  | Std. Deviation | | .726 |  |
|  | Minimum | | 0 |  |
|  | Maximum | | 3 |  |
|  | Range | | 3 |  |
|  | Interquartile Range | | 0 |  |
|  | Skewness | | 2.519 | .243 |
|  | Kurtosis | | 5.998 | .481 |

| **Descriptives** | | | | |
| --- | --- | --- | --- | --- |
|  | | | Statistic | Std. Error |
| **MoCA (/30)** | Mean | | 28.61 | .172 |
|  | 95% Confidence Interval for Mean | Lower Bound | 28.27 |  |
|  |  | Upper Bound | 28.95 |  |
|  | 5% Trimmed Mean | | 28.78 |  |
|  | Median | | 29.00 |  |
|  | Variance | | 2.367 |  |
|  | Std. Deviation | | 1.538 |  |
|  | Minimum | | 23 |  |
|  | Maximum | | 30 |  |
|  | Range | | 7 |  |
|  | Interquartile Range | | 2 |  |
|  | Skewness | | -1.501 | .269 |
|  | Kurtosis | | 2.324 | .532 |

| **Descriptives** | | | | |
| --- | --- | --- | --- | --- |
|  | | | Statistic | Std. Error |
| **Total Scheltens score (/93)** | Mean | | 28.49 | 1.193 |
|  | 95% Confidence Interval for Mean | Lower Bound | 26.12 |  |
|  |  | Upper Bound | 30.86 |  |
|  | 5% Trimmed Mean | | 27.93 |  |
|  | Median | | 28.00 |  |
|  | Variance | | 142.212 |  |
|  | Std. Deviation | | 11.925 |  |
|  | Minimum | | 6 |  |
|  | Maximum | | 73 |  |
|  | Range | | 67 |  |
|  | Interquartile Range | | 13 |  |
|  | Skewness | | .755 | .241 |
|  | Kurtosis | | 1.404 | .478 |

| **Descriptives** | | | | |
| --- | --- | --- | --- | --- |
|  | | | Statistic | Std. Error |
| **White matter hyperintensities (/30)** | Mean | | 9.44 | .594 |
|  | 95% Confidence Interval for Mean | Lower Bound | 8.26 |  |
|  |  | Upper Bound | 10.62 |  |
|  | 5% Trimmed Mean | | 9.07 |  |
|  | Median | | 8.00 |  |
|  | Variance | | 35.299 |  |
|  | Std. Deviation | | 5.941 |  |
|  | Minimum | | 1 |  |
|  | Maximum | | 30 |  |
|  | Range | | 29 |  |
|  | Interquartile Range | | 7 |  |
|  | Skewness | | .992 | .241 |
|  | Kurtosis | | .904 | .478 |

| **Descriptives** | | | | |
| --- | --- | --- | --- | --- |
|  | | | Statistic | Std. Error |
| **Periventricular hyperintensities (/9)** | Mean | | 4.41 | .166 |
|  | 95% Confidence Interval for Mean | Lower Bound | 4.08 |  |
|  |  | Upper Bound | 4.74 |  |
|  | 5% Trimmed Mean | | 4.29 |  |
|  | Median | | 4.00 |  |
|  | Variance | | 2.749 |  |
|  | Std. Deviation | | 1.658 |  |
|  | Minimum | | 1 |  |
|  | Maximum | | 9 |  |
|  | Range | | 8 |  |
|  | Interquartile Range | | 2 |  |
|  | Skewness | | 1.111 | .241 |
|  | Kurtosis | | 1.061 | .478 |

| **Descriptives** | | | | |
| --- | --- | --- | --- | --- |
|  | | | Statistic | Std. Error |
| **Grey matter hyperintensities (/30)** | Mean | | 8.27 | .386 |
|  | 95% Confidence Interval for Mean | Lower Bound | 7.50 |  |
|  |  | Upper Bound | 9.04 |  |
|  | 5% Trimmed Mean | | 8.16 |  |
|  | Median | | 8.50 |  |
|  | Variance | | 14.906 |  |
|  | Std. Deviation | | 3.861 |  |
|  | Minimum | | 1 |  |
|  | Maximum | | 21 |  |
|  | Range | | 20 |  |
|  | Interquartile Range | | 5 |  |
|  | Skewness | | .427 | .241 |
|  | Kurtosis | | .504 | .478 |

| **Descriptives** | | | | |
| --- | --- | --- | --- | --- |
|  | | | Statistic | Std. Error |
| **Infra-tentorial foci of hyperintensity (/24)** | Mean | | 6.36 | .322 |
|  | 95% Confidence Interval for Mean | Lower Bound | 5.72 |  |
|  |  | Upper Bound | 7.00 |  |
|  | 5% Trimmed Mean | | 6.28 |  |
|  | Median | | 6.00 |  |
|  | Variance | | 10.354 |  |
|  | Std. Deviation | | 3.218 |  |
|  | Minimum | | 1 |  |
|  | Maximum | | 15 |  |
|  | Range | | 14 |  |
|  | Interquartile Range | | 4 |  |
|  | Skewness | | .298 | .241 |
|  | Kurtosis | | -.347 | .478 |

**Statistical Analysis**

*ANOVA Total Scheltens Score*

| **Between-Subjects Factors** | | | |
| --- | --- | --- | --- |
|  | | Value Label | N |
| Final diagnosis | 1 | TIA | 17 |
|  | 2 | Minor stroke | 33 |
|  | 3 | Migraine | 25 |
|  | 4 | Non-organic | 7 |
|  | 5 | Other | 18 |

| **Descriptive Statistics** | | | |
| --- | --- | --- | --- |
| Dependent Variable: Lg10 | | | |
| Final diagnosis | Mean | Std. Deviation | N |
| TIA | 1.4140 | .16726 | 17 |
| Minor stroke | 1.4641 | .23291 | 33 |
| Migraine | 1.3893 | .17140 | 25 |
| Non-organic | 1.3029 | .20734 | 7 |
| Other | 1.3936 | .20664 | 18 |
| Total | 1.4129 | .20262 | 100 |

| **Levene's Test of Equality of Error Variances^a,b^** | | | | | |
| --- | --- | --- | --- | --- | --- |
|  | | Levene Statistic | df1 | df2 | Sig. |
| Lg10 | Based on Mean | .905 | 4 | 95 | .464 |
|  | Based on Median | .838 | 4 | 95 | .505 |
|  | Based on Median and with adjusted df | .838 | 4 | 89.570 | .505 |
|  | Based on trimmed mean | .907 | 4 | 95 | .463 |
| Tests the null hypothesis that the error variance of the dependent variable is equal across groups. | | | | | |
| a. Dependent variable: Lg10 | | | | | |
| b. Design: Intercept + Final_diagnosis | | | | | |

| **Tests of Between-Subjects Effects** | | | | | | | | |
| --- | --- | --- | --- | --- | --- | --- | --- | --- |
| Dependent Variable: Lg10 | | | | | | | | |
| Source | Type III Sum of Squares | df | Mean Square | F | Sig. | Partial Eta Squared | Noncent. Parameter | Observed Power^b^ |
| Corrected Model | .192^a^ | 4 | .048 | 1.177 | .326 | .047 | 4.709 | .357 |
| Intercept | 148.060 | 1 | 148.060 | 3632.209 | .000 | .975 | 3632.209 | 1.000 |
| Final_diagnosis | .192 | 4 | .048 | 1.177 | .326 | .047 | 4.709 | .357 |
| Error | 3.872 | 95 | .041 |  |  |  |  |  |
| Total | 203.693 | 100 |  |  |  |  |  |  |
| Corrected Total | 4.064 | 99 |  |  |  |  |  |  |
| a. R Squared = .047 (Adjusted R Squared = .007) | | | | | | | | |
| b. Computed using alpha = .05 | | | | | | | | |

*ANOVA Total Scheltens Scores by Gender*

MALE

| **Between-Subjects Factors^a^** | | | |
| --- | --- | --- | --- |
|  | | Value Label | N |
| Final diagnosis | 1 | TIA | 9 |
|  | 2 | Minor stroke | 19 |
|  | 3 | Migraine | 13 |
|  | 4 | Non-organic | 2 |
|  | 5 | Other | 12 |
| a. Sex = Male | | | |

| **Descriptive Statistics^a^** | | | |
| --- | --- | --- | --- |
| Dependent Variable: Lg10 | | | |
| Final diagnosis | Mean | Std. Deviation | N |
| TIA | 1.3721 | .19460 | 9 |
| Minor stroke | 1.4587 | .24027 | 19 |
| Migraine | 1.4245 | .06442 | 13 |
| Non-organic | 1.4956 | .06853 | 2 |
| Other | 1.3393 | .22319 | 12 |
| Total | 1.4118 | .19625 | 55 |
| a. Sex = Male | | | |

| **Levene's Test of Equality of Error Variances^a,b,c^** | | | | | |
| --- | --- | --- | --- | --- | --- |
|  | | Levene Statistic | df1 | df2 | Sig. |
| Lg10 | Based on Mean | 3.301 | 4 | 50 | .018 |
|  | Based on Median | 2.539 | 4 | 50 | .051 |
|  | Based on Median and with adjusted df | 2.539 | 4 | 39.061 | .055 |
|  | Based on trimmed mean | 3.230 | 4 | 50 | .020 |
| Tests the null hypothesis that the error variance of the dependent variable is equal across groups. | | | | | |
| a. Sex = Male | | | | | |
| b. Dependent variable: Lg10 | | | | | |
| c. Design: Intercept + Final_diagnosis | | | | | |

| **Tests of Between-Subjects Effects^a^** | | | | | | | | |
| --- | --- | --- | --- | --- | --- | --- | --- | --- |
| Dependent Variable: Lg10 | | | | | | | | |
| Source | Type III Sum of Squares | df | Mean Square | F | Sig. | Partial Eta Squared | Noncent. Parameter | Observed Power^c^ |
| Corrected Model | .135^b^ | 4 | .034 | .869 | .489 | .065 | 3.475 | .257 |
| Intercept | 61.010 | 1 | 61.010 | 1568.756 | .000 | .969 | 1568.756 | 1.000 |
| Final_diagnosis | .135 | 4 | .034 | .869 | .489 | .065 | 3.475 | .257 |
| Error | 1.945 | 50 | .039 |  |  |  |  |  |
| Total | 111.697 | 55 |  |  |  |  |  |  |
| Corrected Total | 2.080 | 54 |  |  |  |  |  |  |
| a. Sex = Male | | | | | | | | |
| b. R Squared = .065 (Adjusted R Squared = -.010) | | | | | | | | |
| c. Computed using alpha = .05 | | | | | | | | |

FEMALE

| **Between-Subjects Factors^a^** | | | |
| --- | --- | --- | --- |
|  | | Value Label | N |
| Final diagnosis | 1 | TIA | 8 |
|  | 2 | Minor stroke | 14 |
|  | 3 | Migraine | 12 |
|  | 4 | Non-organic | 5 |
|  | 5 | Other | 6 |
| a. Sex = Female | | | |

| **Descriptive Statistics^a^** | | | |
| --- | --- | --- | --- |
| Dependent Variable: Lg10 | | | |
| Final diagnosis | Mean | Std. Deviation | N |
| TIA | 1.4611 | .12600 | 8 |
| Minor stroke | 1.4715 | .23130 | 14 |
| Migraine | 1.3510 | .23770 | 12 |
| Non-organic | 1.2258 | .19318 | 5 |
| Other | 1.5021 | .11992 | 6 |
| Total | 1.4143 | .21238 | 45 |
| a. Sex = Female | | | |

| **Levene's Test of Equality of Error Variances^a,b,c^** | | | | | |
| --- | --- | --- | --- | --- | --- |
|  | | Levene Statistic | df1 | df2 | Sig. |
| Lg10 | Based on Mean | .803 | 4 | 40 | .530 |
|  | Based on Median | .595 | 4 | 40 | .668 |
|  | Based on Median and with adjusted df | .595 | 4 | 29.468 | .669 |
|  | Based on trimmed mean | .731 | 4 | 40 | .576 |
| Tests the null hypothesis that the error variance of the dependent variable is equal across groups. | | | | | |
| a. Sex = Female | | | | | |
| b. Dependent variable: Lg10 | | | | | |
| c. Design: Intercept + Final_diagnosis | | | | | |

| **Tests of Between-Subjects Effects^a^** | | | | | | | | |
| --- | --- | --- | --- | --- | --- | --- | --- | --- |
| Dependent Variable: Lg10 | | | | | | | | |
| Source | Type III Sum of Squares | df | Mean Square | F | Sig. | Partial Eta Squared | Noncent. Parameter | Observed Power^c^ |
| Corrected Model | .335^b^ | 4 | .084 | 2.033 | .108 | .169 | 8.131 | .556 |
| Intercept | 76.052 | 1 | 76.052 | 1844.434 | .000 | .979 | 1844.434 | 1.000 |
| Final_diagnosis | .335 | 4 | .084 | 2.033 | .108 | .169 | 8.131 | .556 |
| Error | 1.649 | 40 | .041 |  |  |  |  |  |
| Total | 91.997 | 45 |  |  |  |  |  |  |
| Corrected Total | 1.985 | 44 |  |  |  |  |  |  |
| a. Sex = Female | | | | | | | | |
| b. R Squared = .169 (Adjusted R Squared = .086) | | | | | | | | |
| c. Computed using alpha = .05 | | | | | | | | |

*Correlations Total Scheltens Score*

| **Correlations** | | | |
| --- | --- | --- | --- |
|  | | Lg10Sceltens | Age |
| **Lg10Scheltens** | Pearson Correlation | 1 | .550^**^ |
|  | Sig. (2-tailed) |  | .000 |
|  | N | 100 | 100 |
| **Age** | Pearson Correlation | .550^**^ | 1 |
|  | Sig. (2-tailed) | .000 |  |
|  | N | 100 | 100 |
| **. Correlation is significant at the 0.01 level (2-tailed). | | | |

| **Correlations** | | | |
| --- | --- | --- | --- |
|  | | Lg10Scheltns | Lg10MoCA |
| **Lg10Scheltens** | Pearson Correlation | 1 | -.280^*^ |
|  | Sig. (2-tailed) |  | .012 |
|  | N | 100 | 80 |
| **Lg10MoCA** | Pearson Correlation | -.280^*^ | 1 |
|  | Sig. (2-tailed) | .012 |  |
|  | N | 80 | 80 |
| *. Correlation is significant at the 0.05 level (2-tailed). | | | |

| **Correlations** | | | |
| --- | --- | --- | --- |
|  | | Lg10Scheltens | Lg10sBP |
| **Lg10Scheltens** | Pearson Correlation | 1 | .340^**^ |
|  | Sig. (2-tailed) |  | .001 |
|  | N | 100 | 96 |
| **Lg10sBP** | Pearson Correlation | .340^**^ | 1 |
|  | Sig. (2-tailed) | .001 |  |
|  | N | 96 | 96 |
| **. Correlation is significant at the 0.01 level (2-tailed). | | | |

| **Correlations** | | | |
| --- | --- | --- | --- |
|  | | Lg10Scheltens | Lg10dBP |
| **Lg10Schletens** | Pearson Correlation | 1 | .133 |
|  | Sig. (2-tailed) |  | .198 |
|  | N | 100 | 96 |
| **Lg10dBP** | Pearson Correlation | .133 | 1 |
|  | Sig. (2-tailed) | .198 |  |
|  | N | 96 | 96 |

| **Correlations** | | | |
| --- | --- | --- | --- |
|  | | Lg10Scheltens | Lg10PP |
| **Lg10Scheltens** | Pearson Correlation | 1 | .325^**^ |
|  | Sig. (2-tailed) |  | .001 |
|  | N | 100 | 96 |
| **Lg10PP** | Pearson Correlation | .325^**^ | 1 |
|  | Sig. (2-tailed) | .001 |  |
|  | N | 96 | 96 |
| **. Correlation is significant at the 0.01 level (2-tailed). | | | |

| **Correlations** | | | |
| --- | --- | --- | --- |
|  | | Lg10Scheltes | Lg10 RF |
| **Lg10Scheltens** | Pearson Correlation | 1 | .228^*^ |
|  | Sig. (2-tailed) |  | .022 |
|  | N | 100 | 100 |
| **Lg10RF** | Pearson Correlation | .228^*^ | 1 |
|  | Sig. (2-tailed) | .022 |  |
|  | N | 100 | 100 |
| *. Correlation is significant at the 0.05 level (2-tailed). | | | |

| **Correlations** | | | |
| --- | --- | --- | --- |
|  | | Lg10Scheltens | Lg10mRS |
| **Lg10Scheltens** | Pearson Correlation | 1 | .145 |
|  | Sig. (2-tailed) |  | .149 |
|  | N | 100 | 100 |
| **Lg10mRS** | Pearson Correlation | .145 | 1 |
|  | Sig. (2-tailed) | .149 |  |
|  | N | 100 | 100 |

| **Correlations** | | | |
| --- | --- | --- | --- |
|  | | Lg10Scheltens | Lg10NIHSS |
| **Lg10Scheltens** | Pearson Correlation | 1 | .210^*^ |
|  | Sig. (2-tailed) |  | .037 |
|  | N | 100 | 99 |
| **Lg10NIHSS** | Pearson Correlation | .210^*^ | 1 |
|  | Sig. (2-tailed) | .037 |  |
|  | N | 99 | 99 |
| *. Correlation is significant at the 0.05 level (2-tailed). | | | |

*Correlations Individual Scheltens Scores*

WMH

| **Correlations** | | | |
| --- | --- | --- | --- |
|  | | Lg10WMH | Age |
| **Lg10WMH** | Pearson Correlation | 1 | .585^**^ |
|  | Sig. (2-tailed) |  | .000 |
|  | N | 100 | 100 |
| **Age** | Pearson Correlation | .585^**^ | 1 |
|  | Sig. (2-tailed) | .000 |  |
|  | N | 100 | 100 |
| **. Correlation is significant at the 0.01 level (2-tailed). | | | |

| **Correlations** | | | |
| --- | --- | --- | --- |
|  | | Lg10WMH | Lg10MoCA |
| **Lg10WMH** | Pearson Correlation | 1 | -.300^**^ |
|  | Sig. (2-tailed) |  | .007 |
|  | N | 100 | 80 |
| **Lg10MoCA** | Pearson Correlation | -.300^**^ | 1 |
|  | Sig. (2-tailed) | .007 |  |
|  | N | 80 | 80 |
| **. Correlation is significant at the 0.01 level (2-tailed). | | | |

| **Correlations** | | | |
| --- | --- | --- | --- |
|  | | Lg10WMH | Lg10sBP |
| **Lg10WMH** | Pearson Correlation | 1 | .309^**^ |
|  | Sig. (2-tailed) |  | .002 |
|  | N | 100 | 96 |
| **Lg10sBP** | Pearson Correlation | .309^**^ | 1 |
|  | Sig. (2-tailed) | .002 |  |
|  | N | 96 | 96 |
| **. Correlation is significant at the 0.01 level (2-tailed). | | | |

| **Correlations** | | | |
| --- | --- | --- | --- |
|  | | Lg10WMH | Lg10dBP |
| **Lg10WMH** | Pearson Correlation | 1 | .073 |
|  | Sig. (2-tailed) |  | .482 |
|  | N | 100 | 96 |
| **Lg10dBP** | Pearson Correlation | .073 | 1 |
|  | Sig. (2-tailed) | .482 |  |
|  | N | 96 | 96 |

| **Correlations** | | | |
| --- | --- | --- | --- |
|  | | Lg10WMH | Lg10PP |
| **Lg10WMH** | Pearson Correlation | 1 | .352^**^ |
|  | Sig. (2-tailed) |  | .000 |
|  | N | 100 | 96 |
| **Lg10PP** | Pearson Correlation | .352^**^ | 1 |
|  | Sig. (2-tailed) | .000 |  |
|  | N | 96 | 96 |
| **. Correlation is significant at the 0.01 level (2-tailed). | | | |

| **Correlations** | | | |
| --- | --- | --- | --- |
|  | | Lg10WMH | Lg10RS |
| **Lg10WMH** | Pearson Correlation | 1 | .210^*^ |
|  | Sig. (2-tailed) |  | .036 |
|  | N | 100 | 100 |
| **Lg10RS** | Pearson Correlation | .210^*^ | 1 |
|  | Sig. (2-tailed) | .036 |  |
|  | N | 100 | 100 |
| *. Correlation is significant at the 0.05 level (2-tailed). | | | |

| **Correlations** | | | |
| --- | --- | --- | --- |
|  | | Lg10WMH | Lg10mRS |
| **Lg10WMH** | Pearson Correlation | 1 | .169 |
|  | Sig. (2-tailed) |  | .093 |
|  | N | 100 | 100 |
| **Lg10mRS** | Pearson Correlation | .169 | 1 |
|  | Sig. (2-tailed) | .093 |  |
|  | N | 100 | 100 |

| **Correlations** | | | |
| --- | --- | --- | --- |
|  | | Lg10WMH | Lg10NIHSS |
| **Lg10WMH** | Pearson Correlation | 1 | .155 |
|  | Sig. (2-tailed) |  | .127 |
|  | N | 100 | 99 |
| **Lg10NIHSS** | Pearson Correlation | .155 | 1 |
|  | Sig. (2-tailed) | .127 |  |
|  | N | 99 | 99 |

PVH

| **Correlations** | | | |
| --- | --- | --- | --- |
|  | | Lg10PVH | Age |
| **Lg10PVH** | Pearson Correlation | 1 | .467^**^ |
|  | Sig. (2-tailed) |  | .000 |
|  | N | 100 | 100 |
| **Age** | Pearson Correlation | .467^**^ | 1 |
|  | Sig. (2-tailed) | .000 |  |
|  | N | 100 | 100 |
| **. Correlation is significant at the 0.01 level (2-tailed). | | | |

| **Correlations** | | | |
| --- | --- | --- | --- |
|  | | Lg10PVH | Lg10MoCA |
| **Lg10PVH** | Pearson Correlation | 1 | -.202 |
|  | Sig. (2-tailed) |  | .072 |
|  | N | 100 | 80 |
| **Lg10MoCA** | Pearson Correlation | -.202 | 1 |
|  | Sig. (2-tailed) | .072 |  |
|  | N | 80 | 80 |

| **Correlations** | | | |
| --- | --- | --- | --- |
|  | | Lg10PVH | Lg10sBP |
| **Lg10PVH** | Pearson Correlation | 1 | .188 |
|  | Sig. (2-tailed) |  | .066 |
|  | N | 100 | 96 |
| **Lg10sBP** | Pearson Correlation | .188 | 1 |
|  | Sig. (2-tailed) | .066 |  |
|  | N | 96 | 96 |

| **Correlations** | | | |
| --- | --- | --- | --- |
|  | | Lg10PVH | Lg10dBP |
| **Lg10PVH** | Pearson Correlation | 1 | -.033 |
|  | Sig. (2-tailed) |  | .753 |
|  | N | 100 | 96 |
| **Lg10dBP** | Pearson Correlation | -.033 | 1 |
|  | Sig. (2-tailed) | .753 |  |
|  | N | 96 | 96 |

| **Correlations** | | | |
| --- | --- | --- | --- |
|  | | Lg10PVH | Lg10PP |
| **Lg10PVH** | Pearson Correlation | 1 | .277^**^ |
|  | Sig. (2-tailed) |  | .006 |
|  | N | 100 | 96 |
| **Lg10PP** | Pearson Correlation | .277^**^ | 1 |
|  | Sig. (2-tailed) | .006 |  |
|  | N | 96 | 96 |
| **. Correlation is significant at the 0.01 level (2-tailed). | | | |

| **Correlations** | | | |
| --- | --- | --- | --- |
|  | | Lg10PVH | Lg10RS |
| **Lg10PVH** | Pearson Correlation | 1 | .271^**^ |
|  | Sig. (2-tailed) |  | .006 |
|  | N | 100 | 100 |
| **Lg10RS** | Pearson Correlation | .271^**^ | 1 |
|  | Sig. (2-tailed) | .006 |  |
|  | N | 100 | 100 |
| **. Correlation is significant at the 0.01 level (2-tailed). | | | |

| **Correlations** | | | |
| --- | --- | --- | --- |
|  | | Lg10PVH | Lg10mRS |
| **Lg10PVH** | Pearson Correlation | 1 | .150 |
|  | Sig. (2-tailed) |  | .138 |
|  | N | 100 | 100 |
| **Lg10mRS** | Pearson Correlation | .150 | 1 |
|  | Sig. (2-tailed) | .138 |  |
|  | N | 100 | 100 |

| **Correlations** | | | |
| --- | --- | --- | --- |
|  | | Lg10PVH | Lg10NIHSS |
| **Lg10PVH** | Pearson Correlation | 1 | .201^*^ |
|  | Sig. (2-tailed) |  | .046 |
|  | N | 100 | 99 |
| **Lg10NIHSS** | Pearson Correlation | .201^*^ | 1 |
|  | Sig. (2-tailed) | .046 |  |
|  | N | 99 | 99 |
| *. Correlation is significant at the 0.05 level (2-tailed). | | | |

GMH

| **Correlations** | | | |
| --- | --- | --- | --- |
|  | | Lg10GMH | Age |
| **Lg10GMH** | Pearson Correlation | 1 | .399^**^ |
|  | Sig. (2-tailed) |  | .000 |
|  | N | 100 | 100 |
| **Age** | Pearson Correlation | .399^**^ | 1 |
|  | Sig. (2-tailed) | .000 |  |
|  | N | 100 | 100 |
| **. Correlation is significant at the 0.01 level (2-tailed). | | | |

| **Correlations** | | | |
| --- | --- | --- | --- |
|  | | Lg10GMH | Lg10MoCA |
| **Lg10GMH** | Pearson Correlation | 1 | -.153 |
|  | Sig. (2-tailed) |  | .177 |
|  | N | 100 | 80 |
| **Lg10MoCA** | Pearson Correlation | -.153 | 1 |
|  | Sig. (2-tailed) | .177 |  |
|  | N | 80 | 80 |

| **Correlations** | | | |
| --- | --- | --- | --- |
|  | | Lg10GMH | Lg10sBP |
| **Lg10GMH** | Pearson Correlation | 1 | .355^**^ |
|  | Sig. (2-tailed) |  | .000 |
|  | N | 100 | 96 |
| **Lg10sBP** | Pearson Correlation | .355^**^ | 1 |
|  | Sig. (2-tailed) | .000 |  |
|  | N | 96 | 96 |
| **. Correlation is significant at the 0.01 level (2-tailed). | | | |

| **Correlations** | | | |
| --- | --- | --- | --- |
|  | | Lg10GMH | Lg10dBP |
| **Lg10GMH** | Pearson Correlation | 1 | .243^*^ |
|  | Sig. (2-tailed) |  | .017 |
|  | N | 100 | 96 |
| **Lg10dBP** | Pearson Correlation | .243^*^ | 1 |
|  | Sig. (2-tailed) | .017 |  |
|  | N | 96 | 96 |
| *. Correlation is significant at the 0.05 level (2-tailed). | | | |

| **Correlations** | | | |
| --- | --- | --- | --- |
|  | | Lg10GMH | Lg10PP |
| **Lg10GMH** | Pearson Correlation | 1 | .245^*^ |
|  | Sig. (2-tailed) |  | .016 |
|  | N | 100 | 96 |
| **Lg10PP** | Pearson Correlation | .245^*^ | 1 |
|  | Sig. (2-tailed) | .016 |  |
|  | N | 96 | 96 |
| *. Correlation is significant at the 0.05 level (2-tailed). | | | |

| **Correlations** | | | |
| --- | --- | --- | --- |
|  | | Lg10GMH | Lg10RS |
| **Lg10GMH** | Pearson Correlation | 1 | .183 |
|  | Sig. (2-tailed) |  | .069 |
|  | N | 100 | 100 |
| **Lg10RS** | Pearson Correlation | .183 | 1 |
|  | Sig. (2-tailed) | .069 |  |
|  | N | 100 | 100 |

| **Correlations** | | | |
| --- | --- | --- | --- |
|  | | Lg10GMH | Lg10mRS |
| **Lg10GMH** | Pearson Correlation | 1 | .076 |
|  | Sig. (2-tailed) |  | .452 |
|  | N | 100 | 100 |
| **Lg10mRS** | Pearson Correlation | .076 | 1 |
|  | Sig. (2-tailed) | .452 |  |
|  | N | 100 | 100 |

| **Correlations** | | | |
| --- | --- | --- | --- |
|  | | Lg10GMH | Lg10NIHSS |
| **Lg10GMH** | Pearson Correlation | 1 | .207^*^ |
|  | Sig. (2-tailed) |  | .040 |
|  | N | 100 | 99 |
| **Lg10NIHSS** | Pearson Correlation | .207^*^ | 1 |
|  | Sig. (2-tailed) | .040 |  |
|  | N | 99 | 99 |
| *. Correlation is significant at the 0.05 level (2-tailed). | | | |

IFTH

| **Correlations** | | | |
| --- | --- | --- | --- |
|  | | Lg10IFTH | Age |
| **Lg10IFTH** | Pearson Correlation | 1 | .229^*^ |
|  | Sig. (2-tailed) |  | .022 |
|  | N | 100 | 100 |
| **Age** | Pearson Correlation | .229^*^ | 1 |
|  | Sig. (2-tailed) | .022 |  |
|  | N | 100 | 100 |
| *. Correlation is significant at the 0.05 level (2-tailed). | | | |

| **Correlations** | | | |
| --- | --- | --- | --- |
|  | | Lg10IFTH | Lg10MoCA |
| **Lg10IFTH** | Pearson Correlation | 1 | -.193 |
|  | Sig. (2-tailed) |  | .086 |
|  | N | 100 | 80 |
| **Lg10MoCA** | Pearson Correlation | -.193 | 1 |
|  | Sig. (2-tailed) | .086 |  |
|  | N | 80 | 80 |

| **Correlations** | | | |
| --- | --- | --- | --- |
|  | | Lg10IFTH | Lg10sBP |
| **Lg10IFTH** | Pearson Correlation | 1 | .171 |
|  | Sig. (2-tailed) |  | .095 |
|  | N | 100 | 96 |
| **Lg10sBP** | Pearson Correlation | .171 | 1 |
|  | Sig. (2-tailed) | .095 |  |
|  | N | 96 | 96 |

| **Correlations** | | | |
| --- | --- | --- | --- |
|  | | Lg10IFTH | Lg10dBP |
| **Lg10IFTH** | Pearson Correlation | 1 | .054 |
|  | Sig. (2-tailed) |  | .599 |
|  | N | 100 | 96 |
| **Lg10dBP** | Pearson Correlation | .054 | 1 |
|  | Sig. (2-tailed) | .599 |  |
|  | N | 96 | 96 |

| **Correlations** | | | |
| --- | --- | --- | --- |
|  | | Lg10IFTH | Lg10PP |
| **Lg10IFTH** | Pearson Correlation | 1 | .158 |
|  | Sig. (2-tailed) |  | .124 |
|  | N | 100 | 96 |
| **Lg10PP** | Pearson Correlation | .158 | 1 |
|  | Sig. (2-tailed) | .124 |  |
|  | N | 96 | 96 |

| **Correlations** | | | |
| --- | --- | --- | --- |
|  | | Lg10IFTH | Lg10RS |
| **Lg10IFTH** | Pearson Correlation | 1 | .102 |
|  | Sig. (2-tailed) |  | .314 |
|  | N | 100 | 100 |
| **Lg10RS** | Pearson Correlation | .102 | 1 |
|  | Sig. (2-tailed) | .314 |  |
|  | N | 100 | 100 |

| **Correlations** | | | |
| --- | --- | --- | --- |
|  | | Lg10IFTH | Lg10mRS |
| **Lg10IFTH** | Pearson Correlation | 1 | .048 |
|  | Sig. (2-tailed) |  | .633 |
|  | N | 100 | 100 |
| **Lg10mRS** | Pearson Correlation | .048 | 1 |
|  | Sig. (2-tailed) | .633 |  |
|  | N | 100 | 100 |

| **Correlations** | | | |
| --- | --- | --- | --- |
|  | | Lg10IFTH | Lg10NIHSS |
| **Lg10IFTH** | Pearson Correlation | 1 | .127 |
|  | Sig. (2-tailed) |  | .211 |
|  | N | 100 | 99 |
| **Lg10NIHSS** | Pearson Correlation | .127 | 1 |
|  | Sig. (2-tailed) | .211 |  |
|  | N | 99 | 99 |

Multiple Comparison Correction

To control for type I error (i.e. false positives) resulting from multiple comparisons during the above correlations, the FDR method described by Benjamini and Hochberg *et al.* (1995) was applied to all p values by an independent researcher (MS). In the Benjamini-Hochberg procedure, all p values are arranged in ascending order and their critical values are calculated using the formula below:

$$\left( \frac{i}{m} \right)Q$$

*i= rank*

*m=total number of tests*

*Q=FDR threshold*

The next step involves identification of the highest p value that is smaller than its corresponding critical value. All p values above this point (i.e. lower p values) are considered as significant. The table below summarizes this method as applied to our dataset with the point from which p values and above (i.e. lower p values) should be considered as significant highlighted in grey for two different thresholds.

|  |  |  |  | **FDR=0.05** | **FDR=0.1** |
| --- | --- | --- | --- | --- | --- |
|  | **Variable** | **p value** | **Rank** | **(i/m)Q** | **(i/m)Q** |
| Scheltens | Age | 0 | 1 | 0.00125 | 0.0025 |
| PVH | Age | 0 | 2 | 0.0025 | 0.005 |
| WMH | Age | 0 | 3 | 0.00375 | 0.0075 |
| WMH | PP | 0 | 4 | 0.005 | 0.01 |
| GMH | Age | 0 | 5 | 0.00625 | 0.0125 |
| GMH | sBP | 0 | 6 | 0.0075 | 0.015 |
| Scheltens | sBP | 0.001 | 7 | 0.00875 | 0.0175 |
| Scheltens | PP | 0.001 | 8 | 0.01 | 0.02 |
| WMH | sBP | 0.002 | 9 | 0.01125 | 0.0225 |
| PVH | PP | 0.006 | 10 | 0.0125 | 0.025 |
| PVH | RS | 0.006 | 11 | 0.01375 | 0.0275 |
| WMH | MoCA | 0.007 | 12 | 0.015 | 0.03 |
| Scheltens | MoCA | 0.012 | 13 | 0.01625 | 0.0325 |
| GMH | PP | 0.016 | 14 | 0.0175 | 0.035 |
| GMH | dBP | 0.017 | 15 | 0.01875 | 0.0375 |
| Scheltens | RF | 0.022 | 16 | 0.02 | 0.04 |
| IFTH | Age | 0.022 | 17 | 0.02125 | 0.0425 |
| WMH | RS | 0.036 | 18 | 0.0225 | 0.045 |
| Scheltens | NIHSS | 0.037 | 19 | 0.02375 | 0.0475 |
| GMH | NIHSS | 0.04 | 20 | 0.025 | 0.05 |
| PVH | NIHSS | 0.046 | 21 | 0.02625 | 0.0525 |
| PVH | sBP | 0.066 | 22 | 0.0275 | 0.055 |
| GMH | RS | 0.069 | 23 | 0.02875 | 0.0575 |
| PVH | MoCA | 0.072 | 24 | 0.03 | 0.06 |
| IFTH | MoCA | 0.086 | 25 | 0.03125 | 0.0625 |
| WMH | mRS | 0.093 | 26 | 0.0325 | 0.065 |
| IFTH | sBP | 0.095 | 27 | 0.03375 | 0.0675 |
| IFTH | PP | 0.124 | 28 | 0.035 | 0.07 |
| WMH | NIHSS | 0.127 | 29 | 0.03625 | 0.0725 |
| PVH | mRS | 0.138 | 30 | 0.0375 | 0.075 |
| Scheltens | mRS | 0.149 | 31 | 0.03875 | 0.0775 |
| GMH | MoCA | 0.177 | 32 | 0.04 | 0.08 |
| Scheltens | dBP | 0.198 | 33 | 0.04125 | 0.0825 |
| IFTH | NIHSS | 0.211 | 34 | 0.0425 | 0.085 |
| IFTH | RS | 0.314 | 35 | 0.04375 | 0.0875 |
| GMH | mRS | 0.452 | 36 | 0.045 | 0.09 |
| WMH | dBP | 0.482 | 37 | 0.04625 | 0.0925 |
| IFTH | dBP | 0.599 | 38 | 0.0475 | 0.095 |
| IFTH | mRS | 0.633 | 39 | 0.04875 | 0.0975 |
| PVH | dBP | 0.753 | 40 | 0.05 | 0.1 |

**List of Abbreviations**

TIA: transient ischemic attack

dBP: diastolic blood pressure

sBP: systolic blood pressure

PP: pulse pressure

mRS: modified Rankin score

MoCA: Montreal cognitive assessment

NIHSS: national institutes of health stroke scale

WMH: white matter hyperintensities

GMH: grey matter hyperintensities

PVH: periventricular hyperintensities

IFTH: infratentorial hyperintensities

MRI: magnetic resonance imaging

PACS: picture archiving and communication system

ANOVA: analysis of variance

FDR: false discovery rate

**Contributions**

OV: Study design; Participant assessment; Hyperintensities scoring; Statistical analysis; Manuscript write up; Manuscript review.

KT: Statistical analysis; Manuscript write up; Manuscript review.

MS: Study design; Post-hoc statistical analysis; Manuscript review.

CDF: Study design; Post-hoc statistical analysis; Manuscript review.

ADM: Training for hyperintensities scoring; Manuscript review.

CS: Study design; Manuscript review; co-PI.

MJM: Study design; Patient recruitment; Final diagnosis; Manuscript review; PI.

**References**

Easton, J., D., Saver, J., L., Albers, G., W., Alberts, M., J., Chaturvedi, S., Feldmann, E., *et al.* (2009). Definition and evaluation of transient ischaemic attack. *Stroke,* 40, pp. 2276–2293.

Fischer, U., Baumgartner, A., Arnold, M., Nedeltchev, K., Gralla, J., De Marchis, G., M., *et al.* (2010). What is a minor stroke? *Stroke*, 41, pp. 661–666.

Benjamini, Y., & Hochberg, Y. (1995). Controlling the false discovery rate: a practical and powerful approach to multiple testing. *J Royal Stat Soc*, 57, pp. 289–300.

Murray, A. D. (2012). *Brain white matter hyperintensities: correlates in normal ageing and relevance in Alzheimer’s disease* (published online PhD thesis). University of Aberdeen, UK.

Scheltens, P., Barkhof, F., Leys, D., Pruvo, J., P., Nauta, J., J., Vermersch, P., *et al*. (1993). A semiquantative rating scale for the assessment of signal hyperintensities on magnetic resonance imaging. *J Neurol Sci*, 114, pp. 7–12.

Scheltens, P., Erkinjunti, T., Leys, D., Wahlund, L., O., Inzitari, D., del Ser, T., *et al.* (1998). White matter changes on CT and MRI: an overview of visual rating scales. *Eur Neurol*, 39, pp. 80–89.

Varsou, O. (2014). *Neuroimaging of patients with acute focal neurological symptoms: Investigating new functional and structural magnetic resonance imaging measures* (PhD thesis). University of Aberdeen, UK. ISNI: 0000 0004 5360 5409.
